# Experiments Made for the Purpose of Proving That the Small-Pox Is a Disease Common Both to Men and Brutes

**Published:** 1802-09-01

**Authors:** E. Viborg

**Affiliations:** Professor of the Veterinary College of Copenhagen


### *Experiments made for the Purpose of proving that the Small-pox is a Disease common both to Men and Brutes; by* E. Viborg, *Professor of the Veterinary College of Copenhagen.*

In a Treatiſe, entitled, "Experiments and Obſervations on the Effects of different Poiſon in Animals," which I preſented in the year 1792, to the Royal Daniſh Society of Sciences, I communicated ſeveral experiments which I had made by inoculating with the ſmall-pox an aſs, horſe, cow, ſheep, hog, dog, and cat; of which animals, however, the ape was alone infected, and received the ſmall-pox. Notwithſtanding theſe experiments, I remarked, that the tranſition of the ſmall-pox from men to our domeſtic animals ought not to be denied, becauſe Mr. *Barrier*, veterinarian, is ſaid to have obſerved a diſeaſe in dogs, perfectly ſimilar to the ſmall-pox in men. This obſervation by no means aſcertains the identity of both diſeaſes, becauſe that variolous eruption in dogs might have been a peculiar cutaneous diſeaſe, probably originating from a proper infective matter, though it agreed in many reſpects with the real Small-pox. It cannot therefore be proved but by experiments of inoculation, whether the ſmall-pox is a diſeaſe belonging both to men and animals; and only in caſe the ſame infective matter produces in different animals diſeaſes of the ſame nature, we can no longer doubt their real identity. It is known from the obſervations of French phyſicians, that the cow-pox defends the ſheep againſt the ſheep-pox infection, in the ſame manner as they ſecure men from the ſmall-pox, which ſeems evidently to prove the identity of the cow-pox and ſheep-pox; and this obtained an additional proof, by an obſervation made in ſome parts of Denmark, that the ſheep-pox appeared at the ſame time with the ſmall-pox; and it is ſaid to have been likewiſe obſerved in a town of Germany, that hogs received a variolous eruption at the time when the ſmall-pox made their appearance. Theſe obſervations, however, being contrary to my own experiments, and to thoſe of Camper, I purpoſed to repeat them on animals of different kinds. Accordingly I procured matter from malignant ſmall-pox, with which, when ſtill fluid, and only three days old, I inoculated two puppies, by making ſmall inciſions into their ſides with a lancet, and by rubbing in at theſe places the fluid matter. The third day after the inoculation the inciſion had received a red and tumid margin, but no other morbid ſymptom appeared. On the fourth, fifth, and ſixth day the ſwelling and redneſs of the inciſions diſappeared, and and both dogs feemed to be as well as before. On the feventh day, however, red fpots were feen on the naked fkin of the belly and thighs, which on the 8th and 9th days, received in their middle a white puftule, filled with matter, exactly fimilar to the fmall-pox. I was exceedingly furprized at the fuccefs of thefe experiments, as they removed all doubts with refpect to the poffibility of propagating the fmall-pox in dogs, which my former experiments had made me conceive; and I could not account for their not having before fucceeded, though they had been made on four different dogs, with dry as well as liquid matter, the infective power of which was otherwife experienced. The only way of explaining thefe oppofite events of the inoculation, is to fuppofe, that in the firft experiment I accidentally met with dogs that were incapable of receiving the fmall-pox; a circumftance not uncommonly experienced in men, however fingular it may feem, that it fhould have juft happened four times, one after another.

After I had made thefe experiments, I accidentally read in the Medical and Phyfical Journal, Cit. *Marsillac's* notice of a method of inoculation, by giving to two children and to a dog variolous matter fpread between flices of bread and butter, which they ate, and by which means the children, as well as the dog, are faid to have got very mild fmall-pox; an obfervation, which may be alleged as an argument for the above opinion, that the fmall-pox are common to dogs and men. I continued my experiments on a pig, a fheep, and an ape. Although it may appear needlefs to repeat the inoculation on the laft animal, of which it is fufficiently fhewn that it takes the fmall-pox, yet I fhould conclude, from its being infected, that it was not to be afcribed to the matter, if the other animals had not taken the infection. Having, befides, previoufly inoculated the fame ape for the cow-pox, which had only occafioned an eryfipelatous fwelling at the place of inoculation, I wifhed to afcertain, whether this effect of the cow-pox would be fufficient for deftroying the future action of the fmall-pox. June 29th, the ape *, a pig, and a lamb, were inoculated with dry fmall-pox matter, which had been collected on cotton threads five days before.

* The ape taken for this experiment was *Simia innua*. The firft I inoculated was *Simia cynomolgus*; and I am informed, that *Simia sciurea* received the fmall-pox at Amfterdam in the year 1786, by being in the fame room with a child that had the fmall-pox, but without having come in immediate contact with it. It is therefore afcertained of three fpecies of that genus, that they have received the fmall-pox; and from the laft inftance it appears, that they are infected, not only by immediate application of the poifon, but alfo by means of the atmofphere.

Small

Small incisions were made in the arm-pit of the ape, and under the shoulder of the lamb, into which the matter was rubbed in; but in the pig, the thread was drawn through the skin like a seton. In all three animals, the common symptoms of inflammation appeared at the wound on the 4th and 5th day, but particularly the ape had a considerable swelling round the wound, extending to the axillary glands, and which seemed to be very painful. The animal was less lively, but had always a good appetite. On the 6th day after the inoculation the tumour was still the same, and began to receive matter in its middle. The next day variolous eruptions were seen at the chin and right eye, and others appeared by degrees on different parts of the body. These small-pox dried up in the common way, and the 20th day after the inoculation the ape was perfectly recovered, and only a small scab remained on the place of inoculation. The wound made on the pig was not so bad as in the ape; the 7th day, however, the belly was covered with many red small-pox, which having received matter, the next day dried up as usual. The animal shewed no symptom of any internal illness during all this time. In the lamb, the topical symptoms of inflammation disappeared the 6th and 7th day, without having produced the least variolous eruption. I had not expected that the sheep would have remained free from the infection of the small-pox; but though I endeavoured in vain, by repeated experiments, to communicate the small-pox to this animal, yet I strongly suspect that it admits the infection; as it is proved by experiments, made at the Royal Veterinary College at Berlin, that the cow receives the small-pox, which, when inoculated on children, produce again the real small-pox. It would be worth while to try, whether the cow is secured from the infection of the small-pox by a previous infection with the cow-pox. It is likewise interesting to examine the relation between the sheep-pox and the small-pox, to ascertain whether the small-pox are prevented by the inoculation of the sheep-pox, and if the last can be propagated to men, cows, and other domestic animals; whether the cow-pox inoculated to dogs produce the distemper in dogs, as has been intimated by Drs. Jenner and De Carro. But ample is the field which is offered to us by the investigation of the different contagia of animals of their respective relation, and of their capacity of counteracting each other.

